# Surface Alloying and Improved Property of Nb on TC4 Induced by High Current Pulsed Electron Beam

**DOI:** 10.3390/nano11112906

**Published:** 2021-10-29

**Authors:** Xueze Du, Nana Tian, Conglin Zhang, Peng Lyu, Jie Cai, Qingfeng Guan

**Affiliations:** 1 School of Materials Science and Engineering, Jiangsu University, Zhenjiang 212013, China; xueze_du@163.com (X.D.); tianna118@163.com (N.T.); lvp@ujs.edu.cn (P.L.); 2 School of Materials Science and Engineering, Yancheng Institute of Technology, Yancheng 224051, China; 3 Engineering Institute of Advanced Manufacturing and Modern Equipment Technology, Jiangsu University, Zhenjiang 212013, China; caijie@ujs.edu.cn

**Keywords:** high-current pulsed electron beam (HCPEB), TC4 alloy, Nb alloying, microhardness, wear resistance, corrosion resistance

## Abstract

In this paper, an Nb alloying layer on a TC4 alloy was fabricated by using high-current pulsed electron beam (HCPEB) irradiation to improve surface performance. X-ray diffraction (XRD), optical microscopy (OM), laser surface microscope (LSM), scanning electron microscopy (SEM) and transmission electron microscopy (TEM) were used to characterize the phase composition and microstructure of the surface layer. The microhardness, wear tests and corrosion resistance were also examined. The results show that after HCPEB alloying, a Nb-alloyed layer was formed with about 3.6 μm in thickness on the surface of the sample, which was mainly composed of α’-Ti martensite, β-Ti equiaxial crystals, and NbTi_4_ particles. After HCPEB irradiation, the surface hardness, wear resistance and corrosion resistance of Nb alloying layer on TC4 alloy were improved compared to the initial samples.

## 1. Introduction

The TC4 alloy (Ti6Al4V) is widely used in aerospace, military, biomedical and chemical industries because of its good corrosion resistance, high specific strength, stable medium-temperature performance and high biocompatibility [1,2]. However, the TC4 alloy has some unsolved surface problems, including low hardness and poor wear resistance. In addition, TC4 has poor corrosion resistance in some special media (including reducing acid and NaCl solution), which mean it can barely meet the application requirements under some circumstances [3,4]. In order to ensure that the TC4 alloy can give full play to its excellent performance under harsh usage conditions, many researchers have employed the alloying method to treat TC4 alloys by adopting surface treatment techniques [5,6,7]. Despite this, the surface technologies such as vapor deposition, laser surface technology, and ion bean injection have been used widely in the titanium alloys, but some issues including high cost, low efficiency and complex operation remain unsolved [8].

As a new and potential surface alloying technology, HCPEB has been widely studied by scholars for years [9,10]. It is characterized by a large beam spot, strong beam current and short pulse width. The surface layer of the material is injected with the high beam energy to make the surface alloying elements and matrix undergo ultra-rapid heating and cooling at the same time, which leads to the fusion of the alloying elements and matrix and the forming of an alloying layer on the surface of the matrix [11,12,13]. In addition, HCPEB cannot only refine the surface layer of the matrix grain and homogenize the surface composition, but it can also solve the problem of weak interface adhesion between the matrix and the coating by realizing the metallurgical bonding, which is usually unattainable with conventional methods. Many researchers have used HCPEB technology to study surface alloying. For example, Rotshtein [14] adopted an electron beam to alloy Cu onto a 316 stainless steel surface. After that, Zhang [15] conducted a Cr surface alloying treatment on Ni surface, and Yang [16] adopted Cu as an alloying element on a nickel substrate by HCPEB. In the previous works, whether the alloying element could be successfully alloyed depended on the formation of a solid solution or an amorphous phase between the dissolved and the matrix elements. However, Nb is an element with high melting point and good corrosion resistance, which can be easily dissolved in the TC4 alloy, increasing the passivation tendency of the surface and improving the corrosion resistance of TC4. This has important theoretical and practical significance for the service safety of ship parts such as flanges, threads and deep vertical pipes, which need to be served in the marine environment.

In this paper, HCPEB irradiation was used to conduct Nb alloying treatment on a TC4 surface. The influence of the surface element distribution, phase composition and microstructure on the surface properties of the alloying layer under different irradiation was investigated. TC4-Nb surface alloying process and performance improvement mechanisms under the action of HCPEB were discussed.

## 2. Experimental

The annealed industrial TC4 alloy bar was used as the matrix material, and the corresponding chemical composition (wt %) was: Al 6.00%, V 4.30%, Fe 0.16%, C 0.01%, N 0.01%, O 0.18%, H 0.004%, and the balanced Ti. TC4 alloy bars were machined into cylinder with a diameter of 10 mm and thickness of 5 mm, and then they were grounded, mechanical polished, and cleaned ultrasonically in acetone, respectively. The purity of 99.9% Nb powder (400 mesh, particle size about 30~40 μm) was selected as alloying material. The pretreatment is to mix 10 g Nb powder and binder (100 mL, nitrocelluar varnish + diluent, mass ratio 1:2) evenly, then spray it on the mechanically polished TC4 alloy surface with an air-pressurized spraying gun, then dry the sample in a vacuum drying oven at room temperature for 5 h, and the coating thickness measured with a vernier caliper is about 150 μm. Then the “HOPE-I” type HCPEB equipment (DLUT, DL, CHN) was adopted to conduct surface alloying treatment on the pretreated TC4 alloy with 20, 30 and 40 times of pulses. The process parameters of the electron beam are: accelerating voltage 27 kV, energy density of 4 J/cm^2^, a vacuum degree 5.0 × 10^−3^ Pa, a pulse width of 1.5 μs, a beam spot diameter 60 mm, and a pulse interval of 10 s. For more information on the HCPEB system, readers can refer to Ref [17]. Hereafter, the Nb powder coated TC4 alloy after HCPEB irradiation is referred to as an Nb-alloyed sample.

Rigaku D/Max-2500/pc X-ray diffractometer (XRD) (Panalytical, NL)with parameters of CuKα radiation was used to analyze the phase composition of the alloying surface. The morphology of the alloying surface was observed by an ObserverZ1m type Zeiss metallographic microscope (OM), laser confocal microscope (LSM) (OLS4100, JPN), and a scanning electron microscope (SEM) (JEOL JSM-7001F field emission, JPN). The microstructure of the alloying layer was studied bytransmission electron microscopy (TEM) (JEOL-2100, JPN). The sample used for TEM characterization was manually ground on a single surface (the opposite of the irradiated surface), and then the ion mill was thinned to transparency. The microhardness of the sample was measured by microhardness tester (HV-1000, REEDEA, CHN), a load of 0.245 N (25 g) of 15 s was applied to the surface, seven test points were taken from each sample, but the maximum and minimum values were removed to reduce random error. In addition, a 20 min friction and wear test was carried out on an SFT-2M type ball in a testing machine at room temperature, which selected a GCr15 ball (ϕ 6 mm) as grinding materials, and the hardness, the running load, the speed, the turning radius were 62 HRC, 5 N, 300 r/min, 2 mm, respectively. The quality of the sample before and after grinding was measured by an electronic balance. In the electrochemical corrosion experiment, a electrochemical measurement system (CHI760C, Bee Cave, TX, USA) was used, which was the traditional three-electrode system. The 3.5% NaCl solution was a corrosive medium, the platinum net and saturated calomel electrode (SCE) were auxiliary and reference electrodes.

## 3. Results and Discussion

### 3.1. Phase Identification

**Figure 1:**
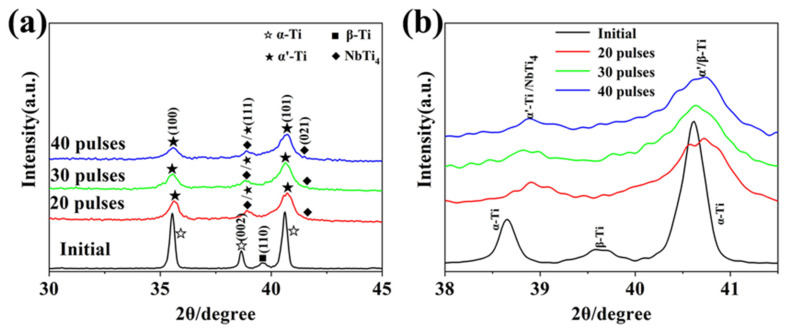
XRD analysis results of the samples before and after Nb alloying. (**a**) XRD patterns; (**b**) Magnified picture of the diffraction peaks near 40°.

Figure 1 shows the X-ray diffraction pattern of initial TC4 alloy and Nb-alloyed samples. It can be seen from Figure 1a that the TC4 alloy is composed of α and β phases. Compared to the original sample (TC4), the location of the α and β phases diffraction peaks showed no obvious difference after 20-pulsed irradiation. Furthermore, the α′ and α phases have the same crystal structure (hcp) and are only slightly different in lattice parameters [18]; they cannot be distinguished by the location of diffraction peak (Figure 1a). But according to related literature [3], the rapid heating and cooling of the high energy beam can make the TC4 alloy undergo martensitic transformation and form α′ martensitic phase due to HCPEB irradiation. Therefore, the diffraction peak at the α phase position should be labeled as α′ phase after alloying, which will be confirmed by TEM analysis in the following. According to the TEM results, the equiaxed β-Ti grains should be formed after HCPEB irradiation, and the β diffraction peak should be seen in the XRD results, so we hypothesized that the β phase should coincide with the α′ phase. Besides, the (101) diffraction peak of α′ phase is widened after HCPEB irradiation, as shown in Figure 1b, indicating the surface grain refinement and lattice distortion increment.

It is worth noting that the NbTi_4_ phase was found on the Nb-alloyed surfaces, which proves that the Nb powders were dissolved into the Ti6Al4V substrate. It confirmed that the surface Nb-alloying on TC4 alloy is successful with HCPEB irradiation.

### 3.2. Surface Topography

**Figure 2:**
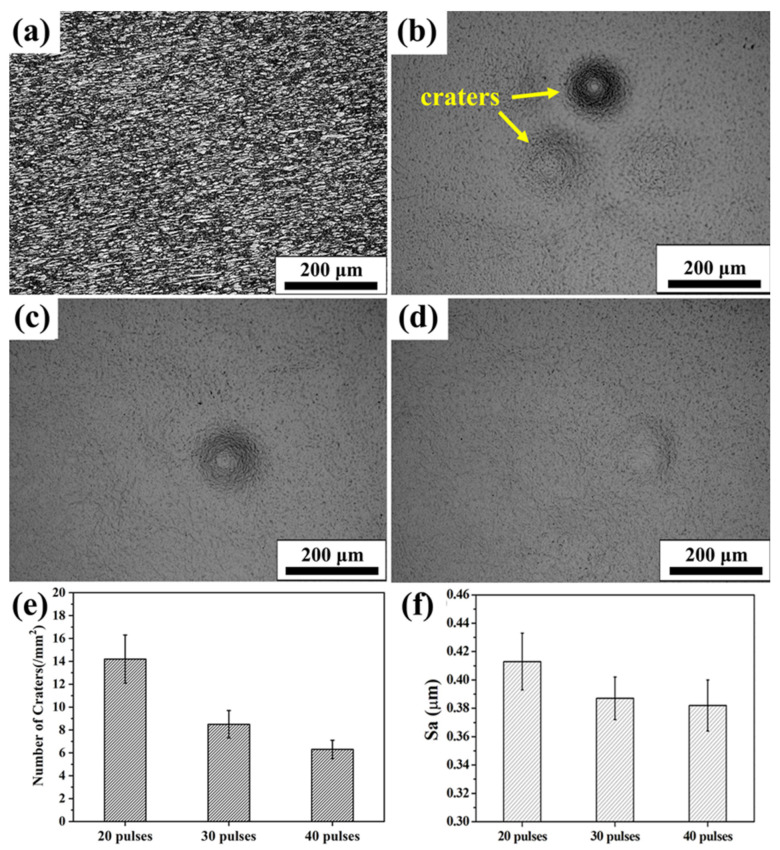
OM images, crater density and surface roughness of initial and Nb-alloyed samples. (**a**) Initial; (**b**) 20 pulses; (**c**) 30 pulses; (**d**) 40 pulses; (**e**) Number of craters; (**f**) Surface roughness.

Figure 2 gives surface morphology, crater density and surface roughness of the initial TC4 and Nb-alloyed samples. The metallographic images of annealed TC4 alloy reveals that the original sample is consists of α (white) and β (black) phases, as shown in Figure 2a. The metallographic morphology (uncorroded) of 20-, 30- and 40-pulsed irradiation samples are shown in Figure 2b–d. The uneven distribution of craters were formed on the irradiated surface, which is consistent with many previous research results [19,20]. Cratering is the most typical morphology characteristic of HCPEB irradiated metal surface, and it is easy to nucleated in the local defects zone, some inclusions or uneven composition of the subsurface layer of the material. Initially, the subsurface layer of the material melts due to the accumulation of energy under HCPEB irradiation. Afterwards the volume of the melted liquid expands rapidly, followed by an eruption from the surface, and then solidifies quickly to form craters [21,22]. The crater density curve indicated that the number of craters decreases gradually with the increase of exposure pulses, as showed in Figure 2e, and the size of craters also decreased. As a result, the surface becomes smooth and uniform. Figure 2f displays the roughness histogram of the sample surface measured by a laser scanning surface microscope after HCPEB irradiation. The surface roughness decreased when the irradiation pulses increased. This is mainly because of the “self-purification” effect of HCPEB irradiation. This can be explained by the fact that a reduction in the number and size of the crater and subsequent fusion of the surface will cause a gradual disappearance in the previously visible formed craters.

**Figure 3:**
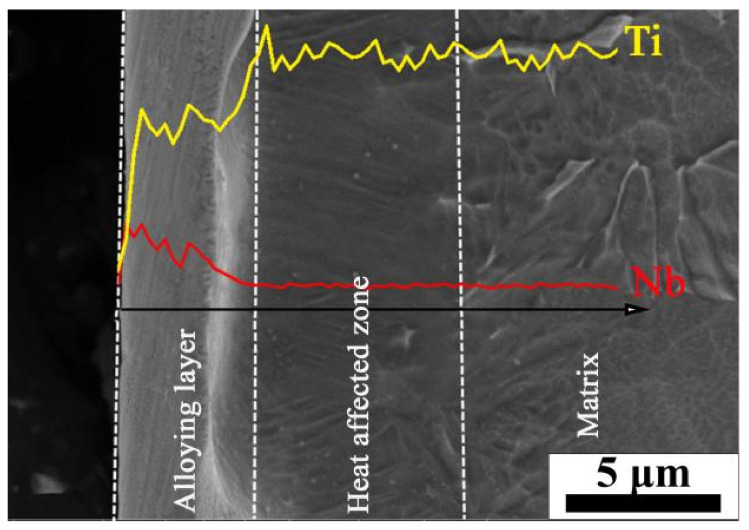
Cross-sectional morphology and EDS analysis of the TC4-Nb samples after 40-pulsed HCPEB irradiation.

The cross-section morphology and EDS line analysis results of 40-pulsed samples are shown in Figure 3. The surface can be categorized into three distinct layers: the alloying layer, the heat affected zone and the matrix. This is basically consistent with the remelting layer of titanium alloy treated by HCPEB irradiation [23]. The EDS line analysis of the horizontal black line revealed that Nb elements only exist within the range of 3.6 µm under the surface. This implied that the Nb elements dissolved into the TC4 matrix to form a Nb-rich alloying layer on the topmost surface after the irradiation. Underneath is the heat-affected zone with the thickness of about 5.7 µm, and its microstructure is homogeneous with fine grains compared to the matrix. The substrate was located below the heat-affected zone and was not affected by HCPEB irradiation.

**Figure 4:**
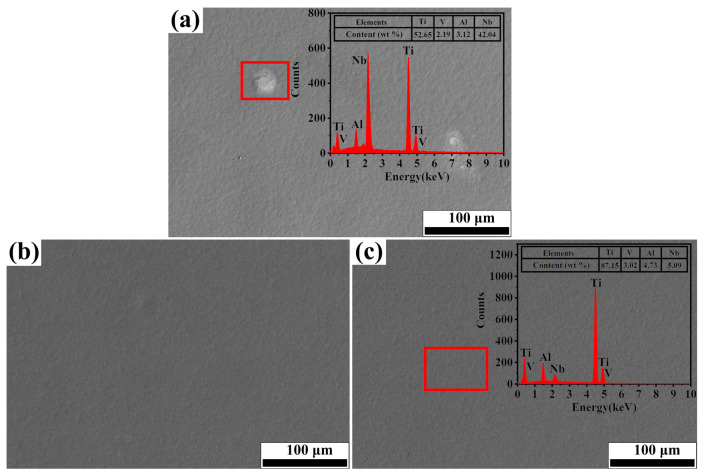
SEM morphology of samples after HCPEB irradiation. (**a**) 20 pulses; (**b**) 30 pulses; (**c**) 40 pulses.

Figure 4 shows the SEM images and EDS results of the Nb-alloyed samples. After 20 pulses of irradiation, no obvious grains can be observed, as shown in Figure 4a. EDS analysis shows that the white area in the red box consisted of the remaining Nb, Ti, Al and V elements. It suggested that the Nb element has not been completely melted into the matrix (Figure 4a). The surface of the samples became even, smooth and uniform when the number of pulses increased from 30 to 40 (Figure 4b,c), and no Nb segregation area was found. The EDS results of the surface for 40-pulsed sample (red box mark in Figure 4c) shows that the Nb content is about 5.0 wt.%, indicating that Nb powder have been melted completely and distributed on the surface with the increase of irradiation pulses.

**Figure 5:**
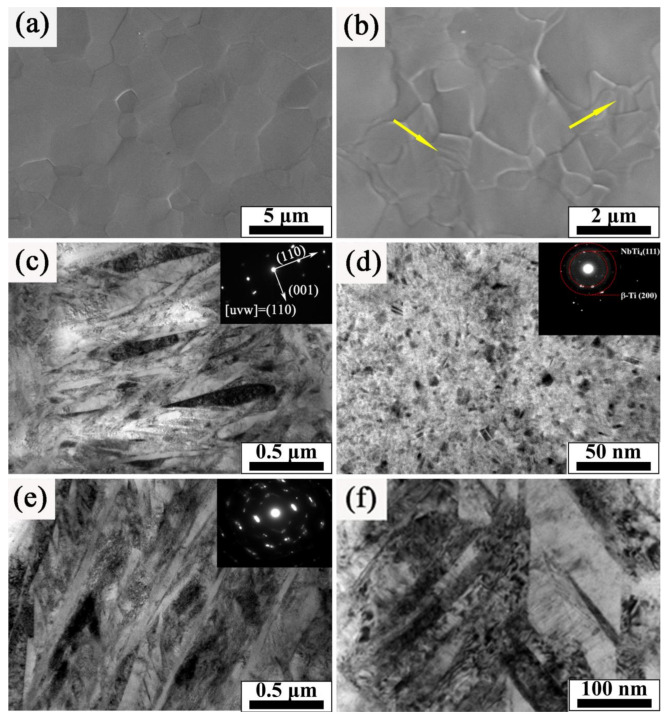
(**a**,**b**) SEM images, (**c**–**f**) TEM images and SAED patterns of the Nb-alloyed sample surface after 40-pulsed irradiation.

Figure 5 illustrates the magnified image of the TC4-Nb surface morphology after 40-pulsed irradiation. From Figure 5a, the Nb-alloyed sample surface consists of equiaxial grains with the size ranging from 1 to 5 μm. Each single individual grain is clearly visible and the grains resulted in an uneven surface. Previous studies demonstrated that the surface morphology was due to the anisotropy of the thermal stress during irradiation, which resulted in the deformation of grains with different orientations [22,24]. The surface morphology at a higher magnification is shown in Figure 5b. It was found that the lamellar structure appears inside the β-phase grain, which was identified as α’ martensite phase according to the XRD results in Figure 1. The formation of α’ phase is due to the rapid solidification of the electron beam and the stress field induced by it, which is consistent with the results in the literature [3,25,26].

Figure 5c–f show the TEM images of the Nb-alloyed samples. Figure 5c gives the lath martensite structure, which was proven to be martensite α′ phase by selected area electron diffraction pattern (SAED). And the equiaxed grains with the width of 50 and 100 nm were found as β-Ti phase. In fact, a β-α’ phase transformation occurs in the β phase during HCPEB. That is, α’ phase is confined within β, while nanocrystals are formed within β. The formation of nanocrystals is related to the rapid cooling of the surface of the matrix, and leads to the widening of β diffraction peak (Figure 1) [3]. For pure titanium, only α’ phase exists on the surface of pure titanium after HCPEB (without β-Ti) [27,28]. In this paper, the addition of Nb prevents the transformation of β-α’ phase, so substantial β-Ti equiaxed grains were formed on the surface of Nb-alloyed samples. This is the same as the result that element C in austenite prevents martensite transformation in carbon steel irradiated by HCPEB [29]. Figure 5d revealed that many fine particles with a size of about 10 nm were formed in the alloying layer, which are NbTi_4_ particles confirmed by SAED pattern. It further proved that Nb was dissolved into the TC4 alloy substrate surface after HCPEB irradiation. In fact, according to the literature [30], NbTi_4_ was observed in an amorphous phase after high-energy ball milling, The morphology of these particles is similar to the NbTi_4_ reinforced particles in this paper. In addition, Banerjee [31] said that Cr_2_Ti can be formed during the laser deposition of the Ti-Cr alloy, so NbTi_4_ is most likely to be formed during the rapid solidification of HCPEB. In this paper, fine NbTi_4_ particles were formed in the Nb-alloyed layer, but with the increase of the number of pulses, the new Nb-alloyed layer continues to melt into the matrix, and part of NbTi_4_ particles will melt into the new Nb-alloyed layer due to surface melting. For this reason, Nb-rich regions will appear near NbTi_4_, which more easily forms equiaxed β grains in the rapid solidification process, This is also why NbTi_4_ particles are found in β grains. In addition, NbTi_4_ particles have the characteristics of dispersion distribution due to the thermal stress caused by HCPEB.

Figure 5e shows that many twin structures were formed in martensite α′ during HCPEB irradiation, and the diffraction spots appeared in pairs. The previous study shows that metal materials with hcp structure tend to form a twins structure during the deformation process, which is mainly due to the lack of sufficient slip system of the hcp structure, thus inducing twinned deformation and the forming a twin structure [32,33,34]. In addition, it was found that the plasticity of metal in the hexagonal system was closely related to the twin structure induced during deformation; the more types and quantities of twins, the better the plasticity of the material [33]. Another common structure in martensite α′ is the high-density dislocation; such a dislocation configuration and even the dislocation wall are often observed after strong plastic deformation of the material, as shown in Figure 5f. Under the action of strong external stress, the higher dislocation density will result in the formation of dislocation tangling. Subsequently, the dislocations were rearranged to form these dislocation walls [35,36]. The formation of a defects structure will have an important influence on the properties of materials such as strengthening.

### 3.3. Microhardness

**Figure 6:**
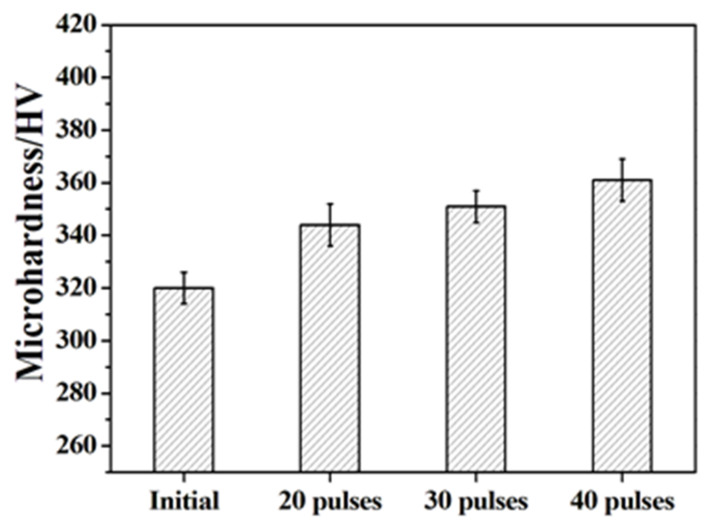
Microhardness measurement of initial and Nb-alloyed samples.

The surface microhardness of the initial and Nb-alloyed samples is shown in Figure 6. The hardness of the original sample was 320 HV. With increasing numbers of pulses, an increment of hardness was found for the Nb-alloyed samples. After 40 pulses, the hardness of the sample rose to 361 HV, which was 12.8% higher than that of the original sample. According to the literature [37], a softened material has been obtained after HCPEB irradiation. The main reason is that HCPEB irradiation induced the formation of sole α′ phase, which is softer than the α-Ti in TC4 alloy. Therefore, the reasons for the improved hardness for Nb alloyed samples has to analyzed.

(1). The temperature gradient of the cooling process was high after HCPEB irradiation, causing the rapid solidification of surface and thereby forming a fine uniform structure. Although the hardness of α′ generated by HCPEB irradiation is lower than that of α martensite, the size of α′ laths was found to be very tiny and accompanied by high density dislocation and twins.
(2). According to Guo’s work [37], the main factor for increasing hardness should be the Nb solid solution strengthening. In this study, the Nb and matrix element diffused each other due to the deposition of the surface energy during irradiation, and it is suggested that the Nb atoms can act as obstacles for the dislocation motion. To illustrate, once the stress required to activate the dislocation increases, the yield increases, leading to a further increase in microhardness, known as solid solution strengthening.
(3). The enhancement of the β phase and the presence of hardening NbTi_4_ phase played a significant role in dispersion strengthening, which is the key to improving the surface hardness of TC4-Nb alloyed samples. In addition, the HCPEB irradiated alloy layer formed an abundant deformation structure (twin crystals and dislocation), which could play the role of splitting martensite, further refining grain, strengthening dislocation and promoting the improvement of surface hardness [5,38].

### 3.4. Wear Resistance

**Figure 7:**
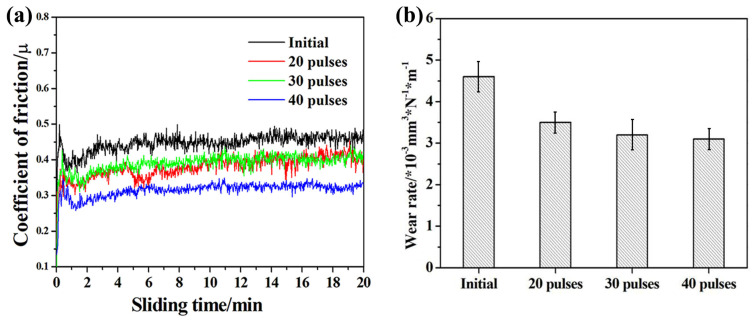
Tribological behavior of initial and Nb-alloyed samples. (**a**) Coefficient of friction; (**b**) Wear rate.

Figure 7 shows the coefficient of friction (COF) and wear rate of initial TC4 and Nb-alloyed samples. Figure 7a demonstrates that the running-in stage of the initial sample is about three min, after which the friction coefficient is stable at 0.48. The COF of Nb-alloyed sample is significantly reduced, and the running-in stages are very short, followed by the stable wear stage. The COF values of Nb-alloyed samples decrease gradually when the pulse number increases and reaches the lowest value after 40 pulses of irradiation, which is 0.32. There is almost no obvious fluctuation in the sliding process, and the COF is relatively stable. The wear rate of Nb-alloyed samples also decreases significantly (Figure 7b), and the relationship between wear rate value and irradiation number is consistent with the COF. In combination with Figure 7a,b, the abrasion resistance of the sample after 40-pulsed irradiation is relatively ideal.

According to he previous study [39,40], the wear mechanism of TC4 could be classified to Equation (1).

(1) $$W=\frac{L}{\nu H}\frac{\beta }{f^{2}{\zeta c}^{2}\;{\rho_{0}}^{2}}$$

For the TC4 alloy, the hardness increases after irradiation, and the wear rate decreases because the abrasion resistance is proportional to hardness. The oxide formed during the dry sliding test of the original TC4 was TiO_2_. But more compact oxide was formed after irradiation, so the *f* and *ρ*_0_ of the irradiated samples were greater than those of the original TC4. In addition, *β* might slightly increased because of the crystal defects and the greater grain boundary density [41].

**Figure 8:**
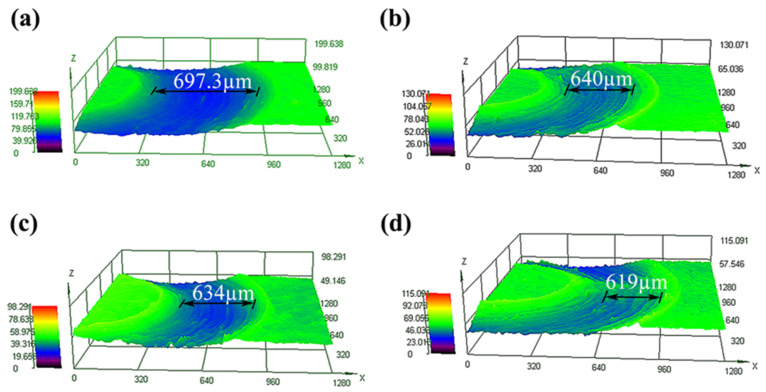
LSM pictures of initial and Nb-alloyed samples. (**a**) Initial; (**b**) 20 pulses; (**c**) 30 pulses; (**d**) 40 pulses.

Figure 8 shows the surface wear profile morphology of the initial and Nb-alloyed samples. A wide wear scratch can be observed from the initial sample, and the wear trace width is about 697.3 µm (Figure 8a). From Figure 8b–d, The width of the wear traces of the 20-pulsed, 30-pulsed, and 40-pulsed irradiation are 640 µm, 634 µm, 619 µm respectively. The values of Nb-alloyed samples are significantly lower than those of the original samples and show a downward trend when the irradiation pulses increased, which are consistent with the results of microhardness and wear resistance. Since the irradiated samples exhibit a lower friction coefficient and wear rate, the surface debris falls off less, which inhibits the damage caused by wear particles, and forms a relatively lower wear width during testing. This is further confirmation that the irradiated sample surface shows excellent tribological properties in the friction and wear test. The improvement in tribological behavior is mostly due to the fact that the Nb-alloyed samples have better adhesion with the substrate than that of the initial samples [37], and the oxide layer formed on the surface of the irradiated sample during friction can also be used as a protective film or lubricant [42]. Therefore, the abrasion resistance of Nb-alloyed samples is better than that of the initial TC4 alloy.

### 3.5. Corrosion Resistance

**Figure 9:**
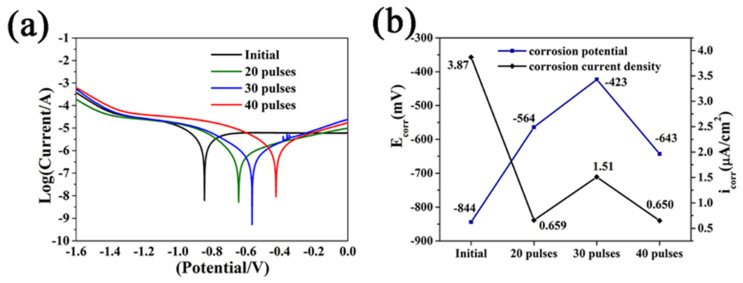
Corrosion performance of initial and irradiated samples in 3.5 wt.% NaCl solution (**a**) Electrochemical corrosion polarization curve; (**b**) Corrosion potential (Ecorr) and corrosion current density (icorr).

The polarization curve, corrosion potential and current density statistics of initial and Nb-alloyed samples in 3.5 wt% NaCl solution are shown in Figure 9. From the electrochemical corrosion polarization curve in Figure 9a, the initial passivation area of the Nb-alloyed samples is significantly large, indicating that the corrosion resistance has been improved after irradiation [43,44]. The current peak appeared in the second passivation area of the irradiated samples because of the continuous self-repair of the oxide film, which contributed to the improved corrosion properties [45].

Figure 9b is the statistical diagram of corrosion potential (E_corr_) and corrosion current density (i_corr_). The E_corr_ of the Nb-alloyed samples was increased, and the most obvious increase was 421 mV after 30-pulsed irradiation. It is difficult to judge the corrosion resistance from a single corrosion voltage because it only reflects the thermodynamic possibility and directivity of metal corrosion. Figure 9b also shows the corrosion current density, which was significantly reduced after HCPEB irradiation. The i_corr_ decreased from 3.87 μA/cm^2^ to 0.659 μA/cm^2^ after 20-pulsed irradiation. The i_corr_ of the 30-pulsed ones is slightly higher, but much lower than that of the original samples, which is 1.51 μA/cm^2^. The corrosion current density reached the lowest value (0.650 μA/cm^2^) after 40-pulsed irradiation, indicating that the anti-corrosion performance was improved.

**Figure 10:**
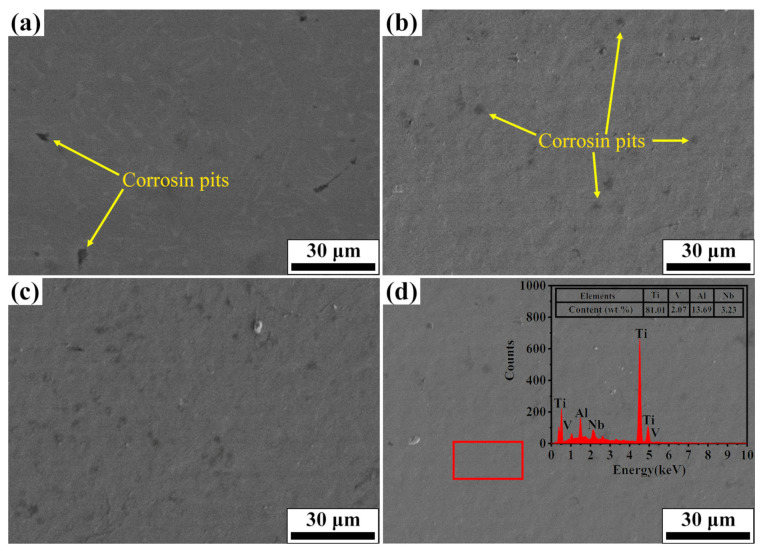
Corrosion morphology and EDS analysis of initial and Nb-alloyed samples. (**a**) Initial; (**b**) 20 pulses; (**c**) 30 pulses; (**d**) 40 pulses.

Figure 10 depicts the corrosion morphology and EDS analysis of initial and Nb-alloyed samples. In Figure 10a, deep corrosion pits and clear edges were found on the initial TC4, implying a severe pitting corrosion, but few corrosion pits were seen on the Nb-alloyed surface after 20, 30, and 40 pulses of irradiation, as observed in Figure 10b–d. With the increase of irradiation pulses, the number and size of corrosion pit are reduced. In addition, EDS analysis of 40-pulsed irradiation in Figure 10d shows that the main elements of the surface are Ti, Al, Nb and O elements, indicating that a uniform protective oxidation film (Nb_2_O_5_) was formed, which acts as protection, reducing the possibilities of pitting corrosion. The reasons for the improvement of corrosion resistance are discussed as follows.

(1). The addition of Nb promotes the nucleation of Nb_2_O_5_ on the surface of the alloy. According to the literature [46,47], when the oxides are formed in the corrosive medium, the oxides are embedded in the matrix of the alloy as independent clusters. When alloying elements are uniformly distributed in each component phase of the alloy, these oxide clusters can be uniformly distributed in the matrix of the alloy, thus forming a stable passivation film on the surface of the alloy, so that the alloy shows good corrosion resistance.
(2). The grains within the Nb-alloyed layer were refined during the irradiation process. The high increase in the number of grain boundaries provided more channels for the rapid diffusion of atoms, which results in the formation of denser oxide film on the top layer of the sample. Moreover, the melting pit and the composition uniformity on the surface have important effects on the compactness of the oxide film [48].
(3). Phase transition of the irradiated sample surface will also affect the corrosion resistance. Previous research showed that the formation of martensite is helpful to improve the corrosion resistance of the samples [44,49]. To sum up, the improvement of surface corrosion performance of Nb-alloyed samples is the combined effect of the above factors.

In addition, the corrosion performance varies with the number of pulses. This is because the number of craters decreases with the increase of the number of pulses (as shown in Figure 2), the channels invaded by Cl^−^ are reduced to improve corrosion performance.

## 4. Conclusions

In this paper, the Nb alloying layer was successfully prepared on the TC4 substrate by different pulses of HCPEB irradiation, and the surface performance was improved. The main conclusions are listed as follows:

(1). Extremely rapid heating, cooling and solidifying during HCPEB irradiation make Nb powder to dissolve into TC4 matrix and form an alloying layer on the surface of the TC4 alloy. A typical pit structure appeared on the surface layer, and the density and roughness of the pit decreased with the increase of irradiation pulses.
(2). Martensitic transformation occurred on the surface of TC4 alloy during HCPEB irradiation, forming a fine strip α′ martensite phase in some β phase grains. Many NbTi_4_ particles with about 10 nm in size were formed in the β phase.
(3). The microhardness and wear resistance of the sample surface were improved, which was mainly due to the joint action of several strengthening mechanisms such as solid solution strengthening, dispersion strengthening and fine crystal strengthening.
(4). The corrosion resistance of TC4-Nb alloys was significantly improved after irradiation, which is the result of Nb alloying, grain refining and surface homogenization.

## Data Availability

All data included in this study are available upon request by contact with the corresponding author.

## Abbreviations

W	wear rate of material
H	hardness
f	fraction of oxygen in oxide
$\rho_{0}$	density of oxide
L	sliding velocity
β	oxidation rate constant
ζc	critical thickness of oxide layer
